# Towards a FAIR metadata framework for drone and uncrewed aerial vehicle data

**DOI:** 10.1038/s41597-025-06376-9

**Published:** 2025-12-08

**Authors:** Florian J. Ellsäßer, Alice Nikuze

**Affiliations:** 1 Faculty of Geo-Information Science and Earth Observation (ITC), University of Twente, Enschede, Netherlands; 2 Library, ICT Services & Archive (LISA), University of Twente, Enschede, Netherlands

**Keywords:** Geography, Research data

## Abstract

Uncrewed Aerial Vehicles (UAVs), or drones, are increasingly used in research areas such as precision agriculture, environmental monitoring, and disaster response. They can carry a wide range of sensors and users produce diverse datasets that include raw imagery, orthomosaics, and spatial models. Sharing these datasets in a Findable, Accessible, Interoperable, and Reusable (FAIR) way requires rich metadata. However, current practices often lack essential details about sensors, processing steps, and licensing, which limits FAIR compliance. We assessed how UAV data is currently published by reviewing metadata from 71 datasets in public repositories. We also evaluated existing metadata frameworks and surveyed over 70 UAV data users and experts to understand their needs and challenges. Based on this analysis, we identify key metadata requirements across the UAV data lifecycle, including information on sensors, spatial and temporal coverage, processing workflows, and provenance. Our goal is to clarify and summarize these needs rather than propose a formal standard. More consistent metadata practices will support the FAIR principles and improve UAV data sharing and reuse in science.

## Introduction

Uncrewed Aerial Vehicles (UAVs), commonly referred to as drones, have seen a growing number of scientific applications in recent years in a wide range of areas, from agriculture, forestry, environmental monitoring to urban planning and disaster and rescue applications^1–6^. The technology has become cheap, easy to use and is available to a very large community of scientists. While most scientific use cases apply UAVs for mapping tasks^7–10^, other applications such as tracking wildlife^11^ or livestock^12^, gathering information on traffic^13^ or the atmosphere^14^ are frequent uses of this versatile technology.

UAVs are considered an ideal platform for spatial research in spatially limited areas and high-resolution mapping scenarios since they offer great flexibility in mission design, allowing users to adjust utilized sensors, flight height, image overlap, point density or sensor angles and many more parameters^1^. Passive optical sensors such as RGB, multi-, hyperspectral and uncooled thermal cameras are the most common payload of UAVs^3,6,15,16^, while active sensors such as LiDAR are becoming increasingly more affordable and available^17–19^. Additional sensors placed in the study area or onboard the UAV might record meteorological data such as relative humidity, cloud cover, incoming solar radiation or other environmental data such as particles, insects, e-DNA or water samples^14,20–22^. This broad availability of sensing options creates a fruitful ecosystem for research, but it also results in a vast diversity of datasets and related descriptive metadata. These datasets can include various levels of processing in the typical lifecycle of UAV-recorded data. Many datasets include the collected raw data^23–27^, such as raw images^24,27,28^ and atmospheric measurements^29,30^ or others, the processed and adjusted data, such as images corrected for atmospheric influences or geotagged data^31,32^. Some datasets encompass first products of processing, like orthomosaics or digital surface models^6,8,10,26^, or the final results of an analysis, such as indices or results in tabular form^27,33,34^.

However, the growing use of UAVs for data collection, along with the diversity of raw and processed outputs, presents new challenges, particularly in ensuring that UAV data can be shared in a Findable, Accessible, Interoperable, and Reusable (FAIR) manner within the scientific community^35^. The FAIR guiding principles for scientific data management and stewardship, seek to provide a guideline to ensure that research data are properly managed and optimized for maximum utility^35,36^. A key focus of these principles is the generation of rich metadata: information that describes the context, content, quality, condition, and characteristics of published datasets^35,36^. Rich metadata enhances the findability of datasets by enabling search engines and catalogues to index and retrieve them based on detailed descriptors^35^. It supports accessibility by providing clear documentation and standardized formats or protocols that facilitate data access for machines and humans^36^. Interoperability is achieved through the use of common vocabularies and machine-readable structures that make the meaning of data explicit, enabling machines to automatically integrate datasets with other systems and services for a unified, semantically coherent view^36^. Finally, metadata promotes reusability by including information on data provenance, licensing, and processing methods, ensuring that both humans and machines can assess the relevance of the data, understand the conditions for reuse, and correctly attribute its creators^36^.

Many data repositories provide a generic metadata summary in a machine-readable format, while in other cases, authors include metadata in supplementary materials such as README files, PDFs, or compressed archives that are not readily accessible or machine-readable. The information that is stored in the metadata varies, sometimes including details on camera types^10,37^ or the type of UAV that was used^23,25^ as well as file formats and additional context. As a result, existing metadata files for UAV datasets lack standardization, ranging from minimal descriptions to highly detailed documentation of data acquisition and processing. While a standardized approach to metadata would clearly support data discovery, integration, and reuse, no comprehensive framework currently addresses the full UAV data lifecycle.

In this work, we do not propose a new standard. Instead, we aim to move towards a FAIR metadata framework by analysing freely available UAV datasets, reviewing existing standards, frameworks and their applicability, and gathering insights from community practices and expert input via questionnaires. This combined approach provides an initial conceptual framework and offers a grounded understanding of current challenges and requirements, which can inform future standardization efforts and be developed into an ISO19115 Community Profile.

## Results

We present the results of a structured review of UAV datasets published in public data repositories, focusing on metadata quality, completeness, and adherence to the FAIR principles. In parallel, we reviewed existing frameworks and standards for UAV and other remote sensing data and conducted a community survey to gather user and expert perspectives on current practices, challenges, and expectations related to UAV metadata. Based on our combined findings from the dataset and framework review and the survey, we propose a set of metadata requirements specifically tailored for UAV remote sensing data, aiming to address current gaps and enhance their findability, accessibility, interoperability, and reusability.

### Review of existing and published UAV datasets

Our review of 71 UAV datasets revealed notable variability in metadata quality^38^. All reviewed datasets included basic descriptive elements such as title, author, and acquisition year. However, only about two thirds of the datasets offered structured metadata, and even among those, the detail and format varied widely^38^. Metadata was often very limited and not consistently provided in machine-readable formats such as XML, CSV, or JSON^38^.

Sensor information was frequently incomplete^24,39,40^. Roughly one-third of the reviewed datasets either did not specify the sensor model or used only vague terms such as “RGB”^41^ or “multispectral”^42^. In many cases, sensor descriptions were limited to UAV platform brand or type names^25,43,44^, while key technical specifications such as spectral bands, resolution, or field of view were omitted.

Descriptions of data processing levels were inconsistent^38^. While some datasets clearly labelled products such as “orthomosaics”^45–47^ or “raw” images or files^44,48^, others used general terms like “results”^49^ or “images”^42^ without further detail. Some dataset descriptions included documentation of the processing chain in varying detail^28,41,50^. Terminology was often inconsistent (for example, referring to products as “NDVI”^32,51^) with limited explanation of the exact applied methods. Information on calibration and flight parameters was rarely available, making reproducibility difficult.

Licensing practices varied considerably^38^. Many datasets used open licenses such as CC0^43,52,53^, CC BY^45,54,55^, or CC BY-SA^6^, but others applied non-commercial terms (e.g., CC BY-NC-SA^5,26,56^) or repository-specific licenses such as the DANS Licence^10,31,57^ or the 4TU General Terms of Use^9,37^. A few datasets lacked clear licensing information altogether^11,27^.

On a positive note, some datasets included persistent links to related publications, code, or derivative datasets^29,34,40,41^, which supports provenance tracking and data integration. However, this practice was not consistent across the collection.

### Review of existing, related standards and frameworks

To better understand the requirements for a UAV-specific metadata framework, we reviewed existing metadata standards and frameworks used in geospatial, remote sensing, and Earth observation domains. The Minimum Information Framework (MIF) was developed specifically to improve the FAIRness of UAV data^58^. It organizes metadata into four classes (project, flight, dataset, and data point) capturing essential information such as mission objectives, flight paths, sensor specifications, and observational context^58^. However, the MIF does not explicitly define data product levels or processing lineage, limiting its usefulness for tracking transformations from raw UAV data to processed outputs like orthomosaics or classification maps. Its broad scope also reduces metadata granularity, hindering reproducibility in complex or analytical workflows.

While the MIF closely focuses on metadata for data acquisition with UAVs, the NASA Data Processing Levels framework, focuses on the details of processing raw data^59^. This framework is widely used in the satellite remote sensing community to classify data products from raw (Level 0) to fully processed and modelled outputs (Level 4)^59^. Defining processing levels is a general trend across scientific domains beyond remote sensing^60^. Fields such as, geochemistry^61^ and magnetotellurics^62^ have recently developed equivalent frameworks to improve data transparency and traceability in complex processes. This system is useful to indicate the degree of processing and helps users understand the processing level of datasets. For UAV applications, aligning with the NASA Data Processing Levels^59^ and classic remote sensing practices enhances transparency and interoperability by clearly distinguishing raw data, intermediate products, and final outputs. This classification supports better understanding of applied processing methods, improves data provenance, and strengthens documentation of workflows.

The ISO 19115 standard and its updated version ISO 19115-1:2014 provide a comprehensive and widely adopted schema for geographic information, covering metadata elements such as dataset identification, quality, spatial and temporal extent, and distribution^63^. While this standard supports interoperability across platforms and disciplines, its complexity and reliance on (digital) technical implementation tools can be a barrier for non-specialist users. Furthermore, the flexibility in how ISO 19115 is applied can lead to inconsistencies in metadata quality, particularly when used without strict implementation guidelines. ISO 19115-1:2014 defines general metadata elements but does not specifically provide fields for acquisition parameters, instruments, or detailed processing workflows. ISO 19115-2:2019, however, extends the standard to explicitly cover acquisition and processing information, including sensors, instruments, methods, and processing history. For UAV data, ISO 19115-1:2014 in combination with ISO 19115-2:2019 offers a valuable structural foundation. However, in ISO, all four processing stages are represented generically as *processStep* elements, providing some flexibility but not necessarily a clearly defined hierarchy of data readiness or usability like in the NASA Data Processing Levels framework^59^. Crosswalks such as the WorldFAIR Cross-Domain Interoperability Framework (CDIF)^64^ as well as established provenance standards like PROV-O and PROV-DM^65^, can help align it with other domain-neutral standards, supporting cross-disciplinary FAIR compliance. Similarly, the INSPIRE directive, which builds upon ISO 19115 and ISO 19139, establishes standardized metadata requirements for spatial datasets within the European Union^66^. Its focus is on harmonizing data across member states and ensuring compliance with common spatial data infrastructure standards. However, INSPIRE’s core metadata profiles are geared toward broad spatial data themes and do not fully capture UAV-specific elements such as high-frequency temporal sampling, detailed flight characteristics, or mission-specific sensor configurations. To be effective for UAV datasets, INSPIRE-compliant metadata would require additional extensions tailored to airborne observations. Other related initiatives include the W3C DCAT family (including DCAT-AP and GeoDCAT-AP)^67^, which enable dataset cataloguing and discovery across portals, and the SpatioTemporal Asset Catalog (STAC)^68^, are increasingly used to describe and search Earth observation data. These frameworks are primarily designed to enhance dataset findability rather than provide detailed acquisition or processing metadata. As such, while they play an important role in supporting the “F” and “A” of FAIR, they lack the granularity required to document UAV-specific parameters such as flight configurations, onboard sensors, and data processing workflows.

Together, these standards and frameworks highlight the value of structured metadata for reuse, interoperability, and transparency. Yet, their limitations in either precision, applicability and complexity reveal the need for a UAV-specific framework that combines the MIF’s domain relevance, the NASA model’s processing clarity, and the comprehensive structure of ISO 19115 or INSPIRE. While useful as reference points, many frameworks fall short in addressing the unique characteristics of UAV platforms, processing workflows, and data products.

### User and expert community insights of the survey

Despite the growing adoption of UAV technologies, the survey with 71 participants, who fully completed it, revealed that a significant portion of respondents have limited experience with UAV metadata^69,70^. This may be partly explained by the fact that the targeted audience included both experts in UAV data acquisition and use, as well as participants from the broader geospatial, Earth observation, and remote sensing communities^70^. Approximately 40% of respondents indicated they had not yet collected or used UAV data themselves, citing reasons such as lack of access, pending training, or organizational roles that do not involve data acquisition. A few others noted that while they received UAV data from others, they were not involved in its documentation^70^. Among those who have worked with UAV data, only a minority had actively documented or shared metadata in a structured manner. Most respondents reported no awareness of UAV-specific metadata standards, while about one-third had some familiarity but no practical experience^70^. Only a small group (around 15–20%) had used or adapted existing metadata standards such as ISO 19115, INSPIRE, or the MIF; or used mature data infrastructures like NASA’s EOSDIS, which support metadata management but are not themselves metadata frameworks. Many acknowledged that metadata is often stored privately, if at all, and rarely passed along to peers or other data users. Several respondents noted that users often do not ask for or understand metadata, leading to its neglect in practice^70^.

Respondents identified several recurring challenges related to UAV metadata documentation and UAV data sharing^70^:

- Large data volumes were frequently cited as a barrier, particularly in terms of sharing or uploading datasets to platforms.
- Many mentioned missing metadata, especially when data were processed long after acquisition or acquired by someone else. Inconsistent practices around documentation further exacerbated this issue.
- Software-related issues included incompatibility between tools, high software costs, and the lack of standardized formats or automated metadata capture features.
- A few respondents shared concerns around data privacy or institutional restrictions on UAV use, particularly when metadata contained sensitive location or project information.

Other technical issues included positional inaccuracies caused by poorly documented positioning methods, variations across different sensors, and inconsistent inclusion of essential parameters like flight paths, GCP accuracy, or sensor calibration.

### Towards a FAIR metadata framework for UAV data

Based on existing frameworks (MIF, NASA Data Processing Levels, ISO 19115 and extensions, and INSPIRE etc.), empirical analysis of public UAV datasets^38^, and insights gathered from our community questionnaire^69,70^, we identified key metadata components that are important to support the FAIR principles in UAV data. These can be summarized as:

- Descriptive metadata: Title, authors, contact information, keywords, discipline, and license.
- Temporal and spatial metadata: Acquisition time, geolocation or bounding box, and spatial resolution or ground sampling distance.
- Sensor and platform metadata: UAV type and model, sensor type, spectral characteristics, and (field-)calibration details.
- Processing metadata: Description of processing levels, software tools and processes used.
- Provenance and linking: References to related publications, datasets, or projects, including DOIs where applicable.
- Crosswalks such as the WorldFAIR CDIF^64^ can help align UAV metadata with domain-neutral standards to support cross-disciplinary FAIR compliance.

Drawing from these components, we outline a flexible, tiered approach to UAV metadata that can accommodate varying levels of detail from basic FAIR compliance to more comprehensive scientific descriptions in Table 1. Our proposed initial framework builds upon ISO 19115-2 by incorporating a structured, interoperable classification (Levels 0–4) adapted from the NASA data processing levels hierarchy. It supports consistent metadata descriptions through standardized keyword guidance and provides clear mappings and crosswalks to existing frameworks and standards. This approach addresses domain-specific needs and components identified through the questionnaire, existing frameworks and standards and datasets review.

**Table 1:** Key metadata categories and example descriptors for a comprehensive FAIR-aligned UAV metadata framework, designed to support varying levels of detail and accommodate passive optical and other sensor types. Mappings (Crosswalk / References) to selected existing standards and ontologies (ISO 19115-1/-2, CDIF, PROV-O) are provided to ensure interoperability and facilitate future alignment with community and institutional metadata frameworks.

FAIR-aligned UAV Metadata Framework					
Descriptive and Administrative					
Category		Keywords	Crosswalk / References		
Context Information		Title, Version, Authors, Contact Information, Organization, Description	ISO 19115-1: CI_Citation, CI_Responsibility, MD_DataIdentification.abstract; CDIF: citation; PROV-O: prov:Agent		
Access & Licensing		Access Level (Open, Embargoed, Restricted, Metadata-Only), License	ISO 19115-1: MD_SecurityConstraints, MD_Constraints.useLimitations; CDIF: accessRights; PROV-O: prov:Activity (usage constraints)		
Classification		Keywords, Language	ISO 19115-1: MD_Keywords, PT_Locale; CDIF: keywords		
Publishing & Funding		Publisher, Funding, Related Project	ISO 19115-1: CI_Citation; CDIF: funding; PROV-O: prov:Entity (funding source)		
Linked Resources		Resource Title, Resource DOI, References, Data Link, Derived From, Same As	ISO 19115-2: LI_Lineage, Documentation: CI_Citation; CDIF: relatedResource; PROV-O: prov:wasDerivedFrom / prov:alternateOf		
Spatio-Temporal Summary		Year, Location	ISO 19115-1: EX_Extent (includes both geographic and temporal extent); CDIF: spatialCoverage, temporalCoverage		
Format & Structure		Formats of Data (TIFF, GeoTIFF, LAS, etc.)	ISO 19115-1: MD_Format; CDIF: format		
**Scope of Dataset**					
**Category**		**Keywords**	**Crosswalk / References**		
Spatial Characteristics		Spatial Coverage (bounding box), Spatial Resolution (e.g., pixel size)	ISO 19115-1: EX_GeographicBoundingBox / EX_Extent,MD_Resolution.levelOfDetail; CDIF: spatialCoverage		
Temporal Characteristics		Time Coverage, Temporal Resolution (flight frequency)	ISO 19115-1: EX_TemporalExtent; CDIF: temporalCoverage		
Platform, Instrument & Sensor		UAV Type, UAV Brand/Version, Sensor Details, Spectra Recorded	ISO 19115-2: MI_Platform, MI_Instrument, MI_Sensor, MI_Plan; CDIF: instrument; PROV-O: prov:Entity		
**Data Processing Levels**					
**Level**		**Description**	**Keywords**	**Format Examples**	**Crosswalk / References**
Level 0		Raw Data	Raw imagery, unprocessed point clouds, sensor logs	JPEG, PNG, TIFF, RAW, LAS, LAZ, CSV, TXT	ISO 19115-2: LI_ProcessStep; PROV-O: prov:Activity
Level 1		Basic Calibrated Data	Radiometrically calibrated imagery, calibrated point clouds, reflectance data	TIFF (calibrated), GeoTIFF, LAS (calibrated), LAZ	ISO 19115-2: Lineage.LI_ProcessStep; PROV-O: prov:Activity
Level 2		Georeferenced & Mosaicked Data	Orthomosaic, georeferenced image layers, terrain products, DSM, DTM from a single mission	GeoTIFF, TIFF, ASC, IMG	ISO 19115-2: Lineage.LI_ProcessStep; PROV-O: prov:Activity
Level 3		Processed & Analyzed Data	Vegetation indices (e.g., NDVI), classified maps, multi-mission terrain models, land cover maps, site-wide DEM/DSM	GeoTIFF, TIFF, SHP	ISO 19115-2: Lineage.LI_ProcessStep; PROV-O: prov:Activity
Level 4		Synthesized Data & Models	Predictive models, 3D surface reconstructions, crop yield estimates, simulations,	OBJ, FBX, GeoTIFF	ISO 19115-2: Lineage.LE_ProcessStep; PROV-O: prov:Activity

This initial framework provides a foundation for FAIR UAV metadata that is adaptable rather than prescriptive. By covering key aspects of both data acquisition and processing, it supports the full UAV data lifecycle by helping data producers document datasets effectively and enabling robust reuse for research and applications.

## Discussion

This study demonstrates that while datasets collected using UAVs are increasingly being shared through established repositories, completeness of metadata associated with these datasets vary significantly. Datasets with minimal metadata such as title, author, and location lack crucial details on collection and processing, while poor documentation harms reproducibility, reuse, and long-term value^71^.

The absence of established metadata frameworks further complicates the situation, with the findability of datasets being severely restricted. Consequently, we argue that our review of existing UAV datasets may be biased towards those datasets published by individuals who are at least partially aware of the importance of publishing FAIR data in public repositories. This suggests that datasets without comprehensive metadata are less likely to be published in such repositories, further limiting the overall pool of data available for reuse.

Our findings reinforce earlier concerns about the limitations of existing metadata standards. While standards such as ISO 19115-1 and INSPIRE are robust in their structure, they are designed for a broad audience and general applicability^63,66^. ISO 19115-2:2019, in particular, provides an extensive model for documenting data acquisition and processing, including detailed processing steps and instrument specifications. However, while processing activities are generically represented through the *processStep* element, ISO 19115-2, focuses more on describing processes rather than categorizing data readiness stages explicitly. The standard does not explicitly define structured processing levels or data readiness stages, which are often essential in remote sensing workflows, particularly when working with diverse UAV data, to distinguish between raw, calibrated, georeferenced, and derived products. In contrast, more targeted approaches, such as the MIF, provide practical checklists for metadata documentation^58^. However, they fall short in addressing the critical aspect of data processing, which may have as significant an impact on data quality as the acquisition phase itself. The MIF paper explicitly mentions a community-driven approach aimed at UAV users who utilize UAVs for scientific data collection^58^, but not necessarily the same community of traditional remote sensing practitioners who are equally concerned with post-acquisition data processing. Other frameworks, such as NASA’s data processing levels, are designed for the remote sensing community, which typically relies on large institutions like NASA, ESA, or private companies for data acquisition^59^. These frameworks prioritise data processing and fall short in addressing data acquisition and context, further contributing to the disconnect between UAV-specific needs and traditional remote sensing standards.

Building on these observations, our findings underscore a disconnect between the needs of UAV data users and the metadata standards currently in use^58^. This disconnect not only hinders data reuse but also impairs integration across datasets and disciplines^36^.

Several repositories have attempted to lower the barriers to UAV data sharing, but in doing so, often sacrifice metadata richness and long-term traceability. For example, OpenAerialMap.org provides a valuable platform for sharing final UAV-data based products such as orthomosaics, yet it lacks support for persistent identifiers (e.g., DOIs) and comprehensive metadata structures. This platform, while still being a great resource, limits both citability and reliable long-term accessibility of shared datasets.

Our proposed metadata framework responds directly to the inconsistencies identified in current UAV data management practices and metadata frameworks that support sharing. It builds on established standards, including ISO 19115-1 and ISO 19115-2, and incorporates crosswalks to initiatives such as CDIF and PROV-O to ensure interoperability and FAIR compliance. We designed the framework to be simple and user-friendly, focusing on the practical realities and immediate needs of the UAV research community. While this work represents an initial step, we envision it evolving into a formal ISO 19115 Community Profile in the future, balancing rigorous standardisation with usability to encourage adoption by practitioners. While many existing frameworks such as ISO 19115, INSPIRE, NASA data processing levels, and the MIF provide valuable elements, few frameworks combine all aspects of UAV data collection, processing, and practical usability for community adoption. By integrating findings from our empirical review of UAV datasets and responses from our stakeholder survey, we developed a tiered, flexible approach tailored to the specific characteristics and practical realities of UAV-based data collection. This flexible metadata model not only reflects the FAIR guiding principles but does so in a way that acknowledges the varying capacities of data producers. At its core, the framework allows for gradual compliance, enabling contributors to start with basic metadata descriptors while encouraging progression toward full lifecycle documentation similar to the progression model described in Řezník *et al*. (2022)^72^. This tiered structure mirrors the diversity of UAV data practices, ranging from small-scale academic studies to operational deployments by government and private sectors. Ongoing international initiatives, such as Persistent Identifiers for Instruments (PIDS)^73^, which aim to standardize instrument metadata, could also be incorporated to further enhance traceability and interoperability. The integration of data processing levels, from raw (Level 0) to synthesized outputs (Level 4), provides clarity for both data providers and secondary users regarding the transformation lineage of UAV data. This is critical, as our review showed that many public datasets omit or ambiguously report on post-acquisition workflows, which undermines reproducibility and reanalysis^38^. Furthermore, the structure encourages the use of persistent identifiers and metadata linkages such as references to related publications, projects, or earlier versions of datasets. This helps bridge a significant gap between UAV data repositories and formal scholarly infrastructures.

In this context, our framework can be seen not as a prescriptive schema but as a pragmatic metadata scaffold. It is sufficiently structured to promote the FAIR principles while remaining adaptable to evolving technologies and community practices. This tiered and flexible structure allows for gradual expansion and mapping to the full ISO 19115-2 standard, supporting a future transition to a formal Community Profile without compromising current usability. This positions it as a valuable starting point for future standardisation efforts and a candidate for adoption in both institutional repositories and community-driven data portals.

## Methods

We conducted a systematic search for UAV datasets published in public data repositories that provided a persistent identifier in the form of a DOI (Digital Object Identifier), ensuring that the datasets are permanently accessible, uniquely identifiable, and citable. Our search covered established data platforms such as DANS, 4TU.ResearchData, Science Data Bank, DRYAD, DataOne, figshare and Zenodo. Platforms such as OpenAerialMap.org were omitted because the data is not connected to a DOI and can be uploaded or deleted by users at any time. This lack of permanence and scholarly traceability makes the datasets unsuitable for inclusion in our review. We used search combinations of keywords such as “drone,” “UAV,” “UAS,” “dataset,” and “metadata.” To supplement repository searches, we performed broader internet searches using engines like Google, DuckDuckGo, Bing, and Perplexity to identify additional publicly accessible UAV datasets. Our inclusion criteria were: (1) the dataset must have a DOI; and (2) metadata in minimal form must be available (title, authors, year), (3) data sets must be stored in an online repository.

### Metadata review and categorization

We gathered metadata from the selected datasets, focusing on basic information such as title, authors and identifier^38^. Each dataset’s metadata was further reviewed and categorized according to several key dimensions: the platform on which the data was stored, the application domain, the type(s) of sensors and UAVs used, the level of processing (ranging from raw data to processed products and analysis outputs), licensing terms and whether the metadata was machine-readable i.e. structured and formatted in a way that enables automatic parsing, interpretation and processing by computer systems^35,74^. Machine-readability in the FAIR context means that data and metadata must be structured in a way that enables computers to autonomously access, interpret, and process them without human intervention, using widely accepted formats, although not necessarily RDF or Semantic Web technologies^74^. We considered both contextual metadata (e.g., title, author, location) and content metadata (e.g., sensor models, processing steps).

To assess the metadata and its alignment with the FAIR principles, we evaluated a total of 71 datasets for the presence or absence, completeness structure and format of its metadata. This included an examination of whether key information was provided to support data discovery, integration, and reuse, such as detailed sensor specifications, descriptions of the processing steps applied to the data, and clarity on licensing. We manually inspected each dataset’s landing page and associated metadata files and recorded key attributes in a structured table^38^. Specifically, we assessed (1) the storage platform (e.g., 4TU.ResearchData), (2) author and publication year, (3) sensor types (e.g., RGB, multispectral, or hyperspectral), (4) application domain, (5) type and processing level of the data (e.g., raw images, orthomosaics, derived products), (6) metadata availability, (7) whether the metadata was in a machine-readable format (e.g., XML, CSV, JSON), and (8) licensing terms. Metadata was classified as either present or absent/limited.

### Review of related existing metadata frameworks

To inform the development of a UAV-specific metadata framework, we reviewed established standards and frameworks commonly used in geospatial and environmental sciences, including ISO 19115^63^ and its extensions, the INSPIRE directive^66^, UAV-oriented initiatives such as the MIF^58^, remote sensing processing-specific standards like NASA’s Data Processing Levels^59^, crosswalk mapping structures such as the WorldFAIR CDIF^64^ and provenance standards like PROV-O and PROV-DM^65^. The review focused on identifying commonalities and divergences among these frameworks, particularly in the types of metadata elements recommended and the extent to which they address UAV-specific acquisition and processing details.

### User and expert survey

To complement this review and assess current practices, familiarity, and challenges related to UAV data and metadata, we designed an online questionnaire titled *“Survey: UAV Data & Metadata Standards”*^69^. The survey aimed to capture participants’ professional roles, experience with UAV data, awareness and use of FAIR data principles, and views on essential elements for a UAV metadata framework.

The questionnaire combined closed- and open-ended questions and was structured into sections covering respondent background, understanding of the FAIR principles, UAV data experience, awareness of existing metadata frameworks and standards, preferred metadata elements, and perceived barriers and enablers to adoption^69^. It was distributed via LinkedIn, online learning platforms, email groups and other academic and professional networks to reach a broad audience of researchers, practitioners, and data specialists in geospatial, remote sensing, and UAV-related fields. Responses were collected anonymously and analysed to identify trends in documentation practices and community needs for improved metadata standardisation.

Based on the analysis of metadata from existing UAV datasets and established standards from related fields, we identified key elements that are important for enhancing the FAIR compliance of UAV data. Drawing on these insights, we outline a set of metadata requirements that aim to balance the need for comprehensive documentation with the practical realities faced by data producers.

Rather than defining a definitive standard, this work contributes to ongoing discussions around what a UAV metadata framework could look like in practice.

## Data Availability

All materials supporting this study are publicly available through the 4TU.ResearchData repository. The replication material includes: 1. Table with reviewed datasets^38^. A curated list of UAV-related datasets analysed in this study, including their metadata completeness and source information. 10.4121/d845f33d-e199-4c96-8a1f-1db2ad9f2a9c 2. User and expert survey questionnaire^69^. The survey instrument used to collect user and expert feedback on UAV metadata practices. 10.4121/e469b017-e6e1-493d-8b8e-60f97cb55ceb 3. User and expert survey results dataset^70^. Anonymized responses from the user and expert surveys, summarized for analysis. 10.4121/4735b645-b103-4f6a-8165-03f59b748b3b
